# Bone-Targeted Therapies in Metastatic Castration-Resistant Prostate Cancer: Evolving Paradigms

**DOI:** 10.1155/2013/210686

**Published:** 2013-08-28

**Authors:** Joelle El-Amm, Ashley Freeman, Nihar Patel, Jeanny B. Aragon-Ching

**Affiliations:** ^1^Division of Hematology/Oncology, Department of Medicine, George Washington University Medical Center, 2150 Pennsylvania Avenue NW, Washington, DC 20037, USA; ^2^Department of Medicine, George Washington University Medical Center, Washington, DC 20037, USA

## Abstract

Majority of patients with metastatic castrate resistant prostate cancer (mCRPC) develop bone metastases which results in significant morbidity and mortality as a result of skeletal-related events (SREs). Several bone-targeted agents are either in clinical use or in development for prevention of SREs. Bisphosphonates were the first class of drugs investigated for prevention of SREs and zoledronic acid is the only bisphosphonate that is FDA-approved for this indication. Another bone-targeted agent is denosumab which is a fully humanized monoclonal antibody that binds to the RANK-L thereby inhibiting RANK-L mediated bone resorption. While several radiopharmaceuticals were approved for pain palliation in mCRPC including strontium and samarium, alpharadin is the first radiopharmaceutical to show significant overall survival benefit. Contemporary therapeutic options including enzalutamide and abiraterone have effects on pain palliation and SREs as well. Other novel bone-targeted agents are currently in development, including the receptor tyrosine kinase inhibitors cabozantinib and dasatinib. Emerging therapeutics in mCRPC has resulted in great strides in preventing one of the most significant sources of complications of bone metastases.

## 1. Introduction

**Figure 1:**
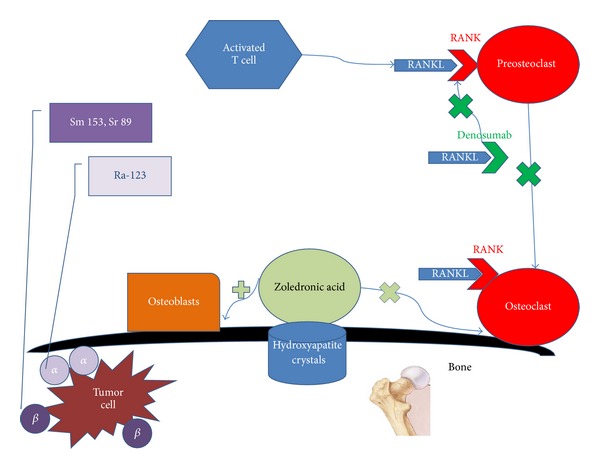
Simplified figure of selected bone-targeted therapies in mCRPC and their targets. Zoledronic acid binds to hydroxyapatite crystals preventing the activity of osteoclasts and stimulating osteoblast. Denosumab binds to RANKL preventing the binding of RANKL to RANK thus inhibiting activation of osteoclasts. Radiopharmaceuticals emit *α* or *β* ionizing radiation to the tumor cell in the bone.

Prostate cancer remains the most common noncutaneous cancer among American men [1]. More than 90% of patients with metastatic castrate resistant prostate cancer (mCRPC) develop bone metastases which results in a significant increase in the risk of morbidity and mortality [2, 3]. The extent of bone involvement in mCRPC has been also found to be associated with patient survival [4]. While most patients are clinically asymptomatic, those with symptoms may manifest with either pain or as skeletal-related events (SREs). SREs are defined variably but typically include manifestations of spinal cord compression, pathological fractures, hypercalcemia of malignancy, requirement for interventions such as bone surgery, or need for bone radiation. Historically, in the absence of bone-targeted therapy, the rate of SREs at 15 months was reported to be 44%, including a 22% rate of fracture [5, 6]. While the mechanisms and lesions in mCRPC have traditionally been thought of as osteoblastic, increasing evidence lends credence to the importance of osteolytic and proosteoclastogenic factors in prostate cancer metastases, which brings about evidence of both an osteolytic and an osteoblastic component with increased bone formation and resorption [7]. Docetaxel, the standard first-line chemotherapy agent in mCRPC, not only improves overall survival (OS) but also improves quality of life and significantly reduced pain (35% versus 22% in placebo, *P* = 0.01) [8, 9]. Increasing recognition of the beneficial effects of agents that delay SREs in the absence of objective overall survival has brought about the routine use of bone-targeted agents (see Figure 1 for mechanisms of action). Certainly, with the advent and use of newer treatment agents such as the CYP17 lyase inhibitor abiraterone acetate and anti-androgen enzalutamide, decreased rate of SREs is being reported with targeting of cancer cell proliferation by these selective agents having effect also on pain response [10–15]. This review describes the bone-targeted therapies that are either established or in development in the treatment of mCRPC (see Table 1 for summary of agents).

## 2. Bisphosphonates

Bisphosphonates were the first class of agents investigated for prevention of SREs in patients with mCRPC. Bisphosphonates are pyrophosphate analogues that adhere to hydroxyapatite crystal-binding sites in the bone matrix [22]. Through attachment to binding sites in areas of active resorption, bisphosphonates prevent osteoclast adherence while inhibiting osteoclast progenitor differentiation and survival through stimulation of osteoblasts [23]. Zoledronic acid is currently the only bisphosphonate approved to prevent SREs in patients with metastatic CRPC. The phase 3, randomized, placebo-controlled trial, which led to the United States Food and Drug Administration (FDA) approval, was conducted in a total of 643 patients with CRPC and asymptomatic bone metastases and were randomized to receive intravenous zoledronic acid at 4 mg, 8 mg, or placebo every 3 weeks for 22 cycles [5]. However, the dose was changed to 4 mg for all participants midway due to concern for renal impairment developing in the high-dose group. The primary endpoint of the study was the proportion of patients who develop SREs. Secondary endpoints included time to the first SRE, skeletal morbidity rate, time to disease progression, objective bone response, biochemical markers, and quality of life parameters. The trial met the primary endpoint with results significant for the zoledronic acid arm being associated with a reduced proportion of patients with an SRE (44.2% versus 33.2%; *P* = 0.021). However, there was no significant difference in overall survival, disease progression, performance status, or quality of life. With a follow-up at 24 months, zoledronic acid decreased the risk of SREs by 36% (Relative Risk (RR) = 0.64, *P* = 0.002), increased the time to first SRE by 167 days (488 days versus 321 days, *P* = 0.009), and decreased bone pain (−0.47% difference on the bone pain index at 24 months, *P* = 0.024) as compared to placebo [6]. Studies of other bisphosphonates have not yielded similar results. Two multicenter, randomized, placebo-controlled trials to evaluate efficacy of pamidronate in CRPC failed to show a reduction in SREs in patients with metastatic prostate cancer and bone pain [24], with results of these two studies reported together. A total of 350 patients with CRPC and painful bone metastases were randomized to receive intravenous pamidronate (90 mg) or placebo every 3 weeks for 27 cycles. Pamidronate is less potent than zoledronic acid, which may account for the lack of efficacy observed in these trials. Additionally, the patient population had more advanced metastatic disease at baseline with painful rather than asymptomatic bone metastases. Similarly, a study of clodronate to evaluate efficacy for palliation of symptomatic bone metastases failed to demonstrate significant pain relief in men with CRPC and bone metastases [25]. Although another trial of oral clodronate versus placebo conducted by the Medical Research Council showed a nonstatistically significant favorable bone progression-free survival with the use of clodronate [26], longer term follow-up of the PR05 trial showed overall survival as a secondary endpoint was statistically significant in the men who received clodronate [27], alluding to an inherent antitumor role of bisphosphonates [28].

Bisphosphonates are fairly well tolerated, with adverse effects including flu-like symptoms such as fatigue, myalgias, and fever, particularly with the first infusions in up to 44%, hypocalcemia in 6%, and osteonecrosis of the jaw (ONJ) in 1% of patients. It remains unclear how bisphosphonates bring about ONJ although certain risk factors have been described which include duration of bisphosphonate use, frequency of use, and poor dental hygiene or intervention [29]. Other reports include use of additional therapy such as corticosteroids or potential additive agents [30, 31]. It is therefore imperative to obtain baseline dental consultations prior to initiating bisphosphonates to determine whether major dental procedures need to be undertaken and avoidance of major surgical dental procedures should be observed once bisphosphonates are already started or being given. Bisphosphonate-induced nephrotoxicity limits their use in many cases and requires careful monitoring and dose-adjustment in patients with renal insufficiency [32]. Given the long potential skeletal half-life of bisphosphonate use [33], the optimal duration of bisphosphonate use is unknown and remains an important question to be answered in view of the potential side effects that may be incurred with prolonged use. 

## 3. Denosumab 

Recent evidence has suggested that development of prostate cancer bone metastases entails osteoclastic activity in addition to osteoblastic activity. Conceivably, the most clinically important proosteoclastogenic factor by prostate cancer cells is receptor activator of NF kappaB ligand (RANK-L) [7]. RANK-L is a tumor necrosis family (TNF) member that is expressed on the surface of osteoblasts and is released by activated T cells. When RANK binds to RANK-L, it stimulates osteoclast formation, activation, adherence, and survival, eventually leading to bone resorption [34–38]. RANK-L is counteracted by naturally occurring osteoprotegerin (OPG), another TNF family member that binds and subsequently prevents activation of its single cognate receptor, RANK, thus, making osteoclastic activity dependent on the balance between both RANK-L as well as OPG [39]. Denosumab is a fully humanized monoclonal antibody that binds to the RANK-L thereby inhibiting RANK-L mediated bone resorption. Denosumab was approved by the FDA in November 2010 for prevention of SREs in patients with bone metastases from solid tumors, including those from prostate cancer. In early clinical trials with humans, two phase I trials were conducted with denosumab in cancer patients with breast cancer and multiple myeloma evaluating safety, pharmacokinetics, and pharmacodynamics [40]. Denosumab exhibited nonlinear, dose dependent pharmacokinetics with rapid and prolonged absorption detectable as early as 1 hour post-dose and average maximum concentration between 7 and 21 days postdose. In 2009, results from a phase II trial of denosumab in patients with bone metastases from prostate cancer as well as other neoplasms after intravenous (IV) bisphosphonate (BP) therapy showed fewer patients receiving denosumab experienced on-study SREs than those receiving IV BPs. A total of 111 eligible patients were accrued with entry criteria of histologically confirmed malignancy, >1 bone metastasis, and urinary N-telopeptide (uNTx) levels higher than 50 nmol/L bone collagen equivalents (BCE)/mM creatinine despite ongoing IV BPs [41]. Elevated uNTx level, a marker for bone resorption, has also been shown to be an independent prognostic factor for overall survival in patient with bone metastases from castrate resistant prostate cancer receiving bisphosphonate therapy [42]. To further determine the effects of denosumab on bone mineral density and fractures in men receiving androgen deprivation therapy for prostate cancer, a randomized, double-blinded, multicenter study, known as the HALT prostate cancer trial, assigned men to receive denosumab at a dose of 60 mg subcutaneously every 6 months or placebo, with the primary endpoint, percent change in BMD at the lumbar spine at 24 months. At 24 months, denosumab was associated with increased BMD at all sites including lumbar spine, femoral neck, and total hip, as well as a reduction in the incidence of new vertebral fractures among men receiving ADT for nonmetastatic prostate cancer [43]. This pivotal trial eventually led to the FDA-approval of the use of denosumab for men with nonmetastatic prostate cancer receiving androgen deprivation therapies who are at high risk for developing fractures. Two randomized, double-blinded clinical trials have investigated the efficacy of subcutaneous denosumab in prostate cancer [16, 44]. A phase III, randomized, double-blinded trial comparing denosumab with zoledronic acid for prevention of SREs in men with bone metastases from CRPC was conducted with a total of 1904 patients randomized [16]. Of the 950 patients assigned to denosumab, the median time to first SRE was 20.7 months compared to 17.1 months in the 951 patients assigned to zoledronic acid (*P* = 0.0002 for noninferiority and *P* = 0.008 for superiority, HR 0.82). Adverse events were similar in both groups, though more events of hypocalcemia occurred in the denosumab group than in the zoledronic acid group 13% versus 6%, *P* < 0.0001). This registration trial led to the FDA-approval of denosumab with the indication of prevention of skeletal-related events in men with metastatic prostate cancer. Another subsequent phase III, randomized, double-blinded, placebo-controlled trial, specifically gauging bone-metastasis-free survival in men who are at high risk of developing bone metastasis (i.e., those with a PSA of ≥8.0 *μ*g/L or PSA doubling time of ≤10.0 months, or both), as determined by time to first occurrence of bone metastasis (symptomatic or asymptomatic) or death from any cause [44] was conducted and enrolled 1432 patients who were randomly assigned to treatment groups. Though no difference in overall survival was seen between groups, denosumab was shown to significantly increase bone-metastases-free survival by a median of 4.2 months as well as significantly delay time to first bone metastases compared with placebo. However, these endpoints were not deemed clinically significant enough such that the FDA ruled against approval of denosumab for specific use for this particular indication of delaying bone metastases.

## 4. Radiopharmaceuticals

Bone-seeking radiopharmaceuticals have historically been available but relegated as a palliative treatment for pain in patients with metastatic prostate cancer [45]. Radiopharmaceuticals emit either alpha or beta particles. An alpha particle, which is ejected from a heavy nucleus during alpha decay, consists of two neutrons and two protons. A beta particle is an electron released from a nucleus containing excess neutrons during beta decay, in which one neutron is converted to a proton, an electron, and a neutrino. Both *α*- and *β*-particles can deliver damaging radiation locally to cancerous cells [46]. Several *β*-emitting radiopharmaceuticals (strontium-89, 153Sm-EDTMP, and Re-186 HEDP) are approved for palliation of pain caused by bone metastases from prostate cancer. The most prominent limitation of these agents is myelosuppression. Radiopharmaceuticals are underutilized in clinical practice, mainly because of the concern for significant myelosuppression, the dependency on other subspecialists (i.e., nuclear medicine specialists or radiation oncologists) for administration, and because until results on alpharadin has emerged, no survival advantage was supported by clinical data. 

### 4.1. 89Sr and 153Sm

The most commonly used radiopharmaceuticals, both *β*-emitters, initially approved in the US for treatment of bone metastases are Strontium-89 chloride or 89Sr (Metastron; GE Healthcare, Arlington Heights, IL) and Samarium-153 or 153Sm (Quadramet; EUSA Pharma, Oxford, UK). There was no demonstration of improvement in overall survival in Phase III trials, although palliative benefits were seen that formed the basis of US FDA approval [17–19, 47–50]. Although there is some evidence that these beta-emitting radioisotopes might provide a small benefit with complete reduction in pain over 1–6 months and no increase in analgesic use, severe adverse effects (mainly leukopenia and thrombocytopenia) are relatively frequent [51].

Sr-89 was initially FDA approved in 1993 as the first beta-emitting radiopharmaceutical for metastatic prostate cancer. Sr-89 is a divalent ion that is incorporated into the inorganic matter of bone when injected intravenously, its half-life is 50.5 days with a beta energy of 1.5 MeV, without emission of gamma energy, and is renally excreted rapidly [52, 53]. 

Several studies have investigated the relationship between the dose of Sr-89 and clinical responses in terms of bone pain palliation. A phase I/II study reported mean time-to-onset of response at 9 days with average duration-of-response of 1.6 months in patients receiving doses ranging from 1.0 to 4.0 mCi/kg [54]. In contrast, another study reported no dose-response relationship with increasing Sr-89 doses from 1.5 to 3.0 MBq/kg [52]. A systematic review summarized the efficacy of Sr-89 and reported that complete pain response varied from 8% to 77% with a mean value of 32% [53]. The mean percentage of patients with a partial pain response was 44% with a time delay until the onset of treatment effect varying from 4 to 28 days, with the mean duration of response lasting 15 months. Reduction in analgesic use was between 71% and 81%.

The principal toxicity of strontium-89 is hematologic in nature, with an average reduction in white blood cells (WBC) of 15% and platelet count of 25–45% in patients receiving the recommended dose of 4.0 mCi or 150 MBq [52, 55]. Predicted nadirs occur at around 6 weeks, and count recovery can take up to 6 months.

153Sm conjugated to ethylene-diamine-tetra-methylene-phosphonic acid (EDTMP) was FDA approved in 1997 at a dose of 1 mCi/Kg. The half-life is 1.9 days and pain relief is rapid, generally between 2 and 7 days [18, 56]. Gamma emission is 103 keV, allowing for scintigraphic imaging, and indeed, images strongly correlate with conventional technetium-99 bone scans. However, marrow toxicity remains the principal side effect. Platelet and white cell counts go down between 3 and 6 weeks and generally recover by 8 weeks [19, 56]. Across three randomized trials using a single administration of samarium-153 1.0 mCi/kg, grade 3+ thrombocytopenia was 3–15% and grade 3+ neutropenia was 5–14% [18, 19, 57]. At standard doses, mean platelet reductions were 43–45% and mean WBC declines were 49–51% of baseline [18, 57]. As such, most clinical trials have used hematologic parameter limitations at trial entry. Other contraindications to the use of beta-emitting radiopharmaceuticals include radiotherapy within the previous 2 months, impending cord compression or pathologic fracture, significant renal insufficiency, Karnofsky Performance Status <50%, and disseminated intravascular coagulation.

Since single dose of 153Sm has demonstrated palliative responses, the tolerability of repeated dosing has also been explored. 153Sm can be administered safely and effectively with repeat dosing of 1.0 mCi/kg [20]. In patients receiving two or more doses of 153Sm, time to platelet, or WBC nadir did not change after the first dose. 12% experienced grade 3+ thrombocytopenia and recovery to a platelet count 975,000/mm^3^ occurred by week 8 in 90.4% of patients.

### 4.2. Radium 223

Alpharadin (Radium 223; 223Ra), marketed as Xofigo; Bayer Health Pharmaceuticals, Wayne, NJ, is an *α*-particle emitter with high affinity for the bone matrix and forms complexes with hydroxyapatite at areas of increased bone turnover. *α*-particle emitters deliver a more localized radiation with very short ranges of <100 *μ*m than do *β*-emitters. They have higher mutagenic and lethality potential effects through DNA damage [58]. It is excreted through the gastrointestinal tract with a half-life of 11.4 days and low gamma irradiation [59, 60]. Moreover, it is unique in comparison to beta emitters in that it delivers high linear energy with very small track length (<0.1 mm in tissue) and subsequently far less myelosuppression to the bone marrow. An early phase I trial that included 15 prostate cancer patients examined the feasibility and safety of 223Ra in the treatment of skeletal metastases in prostate and breast cancer patients [60]. The findings showed a remarkable median decline in the serum alkaline phosphatase average of up to 52%. Given the associated pain relief, tolerability, and the rapidity of clearance from the bloodstream, further phase II trial was initiated in men with mCRPC who had pain requiring external beam radiotherapy [59] with promising results leading to the initiation of the global phase III trial ALSYMPCA. 

The ALSYMPCA trial (ALpharadin in SYMptomatic Prostate CAncer) is the first randomized phase III trial to demonstrate improved overall survival with a bone-seeking radioisotope [21]. A total of 922 patients with mCRPC across 19 countries were recruited. All patients were required to have progressed with symptomatic bone metastases with at least 2 metastatic sites on scintigraphy in the absence of visceral metastases. All recruited patients had either received previous docetaxel, refused docetaxel, or were ineligible for docetaxel.

Randomization was 2 : 1 in a double-blind fashion to receive 6 cycles of intravenous 223Ra on a 4-week schedule with best standard of care or 6 infusions of placebo with best standard of care. The trial was halted early after a planned interim analysis found a survival benefit in favor of 223Ra. Updated analysis has demonstrated a 3.6-month survival advantage (14.9 versus 11.3 months, resp., *P* = 0.00185, HR = 0.695). The study therefore met its primary endpoint. In addition, the frequency of skeletal-related events was reduced in the 223Ra group, and the median time to a SRE increased (15.6 versus 9.8 months). Radium-223 is also less toxic than the previous generation of bone-seeking radionuclides. It was well tolerated with low rates of grade 3/4 neutropenia (1.8% versus 0.8%) and thrombocytopenia (4% versus 2%). This trial formed the basis of approval by the FDA of alpharadin on May 15, 2013 for patients with symptomatic mCRPC to the bones in the absence of visceral metastases. The recommended dose and schedule for alpharadin is 50 kBq/kg (1.35 microcuries/kg) administered by slow intravenously over 1 minute every 4 weeks for 6 doses. Given the potential for hematologic toxicity with about 2% of patients in the alpharadin arm sustaining bone marrow toxicity and pancytopenia, certain parameters are required prior to first administration, with absolute neutrophil count ≥ 1.5 × 10^9^/L and, hemoglobin ≥ to 10 g/dL and platelet count greater than or equal to 100 × 10^9^/L. The ability to utilize Radium 223 in the clinic may shift the paradigm with regard to the use of radiopharmaceuticals such that it may truly be a viable treatment option even in men before chemotherapy unlike older radiopharmaceuticals that have usually been relegated to use in the end-of-life care setting. While there are no current guidelines that would dictate optimal sequencing strategies that incorporates the use of radiopharmaceuticals with contemporary agents, the role of radiopharmaceuticals, specifically Radium 223, is anticipated to increasingly gain preference especially in the setting of symptomatic or asymptomatic patients presenting with predominantly bony metastases with the feasibility of continuation of concomitant androgen-biosynthesis inhibitors or antiandrogens. 

### 4.3. Combination with Other Agents

In combining a radiopharmaceutical with chemotherapy to enhance antitumor effects, several phase I/II trials have explored the use of repeated doses of samarium-153 in combination with increasing doses of docetaxel. These trials did not reach dose limiting toxicity [61, 62]. Thus, one can perhaps reap the benefits of one agent known to increase survival (docetaxel) and use this concurrently with a radiopharmaceutical known to improve bone pain, thereby extending life and improving pain. However, the use of combination agents still requires caution, and only in a clinical trial setting. Recent data presented at the 2013 American Society of Clinical Oncology (ASCO) Annual meeting showed feasibility of combining docetaxel and alpharadin, though need for dose reduction of both agents [63].

Some studies have suggested a potential for the combination of radiopharmaceuticals with other systemic therapies [64]. Combination therapy is under study in two notable phase 3 trials. A US National Cancer Institute–sponsored study combines strontium-89 with either docetaxel with prednisone or the ketoconazole, adriamycin, vinblastine, and estramustine regimen (NCT00024167). Similarly, the UK TRAPEZE trial randomized men with CRPC metastatic to bone to receive one of four regimens: (1) docetaxel with prednisolone; (2) docetaxel, prednisolone, and zoledronic acid; (3) docetaxel, prednisolone, and strontium-89; or (4) docetaxel, prednisolone, zoledronic acid, and strontium-89. The rationale behind the trial stems from early data on the use of zoledronic acid which was not widely used in the UK as well as the palliative effects of strontium as well as to achieve a consolidation effect after chemotherapy as a radionuclide [65]. The results of the trial were recently presented at the 2013 ASCO Annual meeting [66]. A total of 757 patients were randomized to one of the four regimens and the primary outcomes of the study were clinical bony PFS which is a composite endpoint of bone pain progression, development of a clinical SRE (no blinded or protocol-mandated radiologic assessment) or death as well as cost-effectiveness, with the former endpoint being reported. Secondary outcomes were SRE-free interval, PSA progression-free survival, toxicity, total SREs, and OS. After 6 cycles of docetaxel, Sr-89 improved CPFS (HR = 0.845, *P* = 0.036). Not surprisingly, no overall survival benefit was seen. While the zoledronic acid arm did not show improved CPFS (as a primary outcome) or OS, it showed improvement in SRE-free interval from 13.1 to 18.1 months whereas the strontium arm did not show statistically significant SRE-free interval. While the findings suggest a potential role of Sr-89 as postchemotherapy maintenance, the specific therapeutic benefit of this radiopharmaceutical may be limited especially in light of the more contemporary radiopharmaceutical with the use of alpharadin that has shown overall survival in addition to traditional SRE effects. 

## 5. Select Agents with Bone-Targeted Effects

### 5.1. Cabozantinib

Cabozantinib (formerly XL-184, Cometriq, Exelixis, San Francisco, CA) is a novel receptor tyrosine kinase inhibitor that inhibits the hepatocyte growth factor c-Met and the vascular endothelial growth factor receptor 2 (VEGFR2), among other pathways. In a phase II randomized discontinuation trial, cabozantinib resulted in partial resolution of bone lesions in 56% of patients and complete resolution in 19% of the patients [67]. These objective responses correlated with pain and bone turnover markers 55% of patients had declines of ≥50% in plasma C-telopeptide, and 56% of patients with elevated total alkaline phosphatase had declines of ≥50% and of the 28 patients receiving narcotics for bone pain, 64% had improvement in pain intensity and 46% were able to decrease or discontinue narcotics. In another dose-finding phase II trial using cabozantinib that looked at 3 varying doses of 60, 40, and 20 mg with a primary endpoint of week 6 bone scan response, defined as ≥30% decrease in bone scan lesion area, the dose of 40 mg was found to be associated with a high rate of bone scan response with better tolerability compared to the 100 mg dose [68]. Whether the radiographic bone responses translate into a survival benefit and durable clinical response will be determined in upcoming phase III trials. The promising results have prompted the phase III study known as COMET-2 (CabOzantinib MET Inhibition CRPC Efficacy Trial) of cabozantinib versus mitoxantrone and prednisone to demonstrate a primary endpoint of pain reduction [ClinicalTrials.gov identifier: NCT01522443]. A separate phase III trial, COMET-1, will assess for OS [ClinicalTrials.gov identifier: NCT01605227]. Cabozantinib is a promising agent given its oral administration, its effect on pain and bone scans, and its unique targeted pathway. 

### 5.2. Dasatinib

Dasatinib (Sprycel, Bristol-Myers-Squibb, Princeton, NJ) is a tyrosine kinase inhibitor that inhibits Src, a mediator of osteoclastic activity, tumor growth, and metastases [69]. In a phase I/II trial of dasatinib combined with docetaxel, 30% (*n* = 14) of patients had disappearance of a lesion on bone scan and another 41% (*n* = 19) had stable bone scans. Bone markers also declined in >75% of patients (87% experienced urine N-telopeptide declines and 76% had decreases in bone-specific alkaline phosphatase levels) [70]. Similarly, a phase II trial with dasatinib monotherapy yielded encouraging activity in the bone with reduction in urinary N-telopeptide in half of evaluable patients with lack of progression in 24 weeks in 43% of patients [71]. Results detected in the bone prompted a phase III, multinational, randomized, double-blinded, placebo-controlled trial (READY) with a primary endpoint of overall survival, and secondary endpoints of SRE and pain. However, this study was recently reported at the Genitourinary Cancers Symposium and showed no difference in overall survival with a median OS of 21.5 months in the combination arm versus 21.2 months in the dasatinib/placebo arm (hazard ratio [HR], 0.99; log-rank *P* = 0.90) [72]. Further analyses of whether the changes in bone markers reflect only bone resorption changes or true tumor dynamic changes are ongoing and recently reported [73].

### 5.3. Abiraterone Acetate

Abiraterone acetate (Zytiga, Janssen/Ortho-Biotech, Horsham, PA) is an inhibitor of CYP17 that functions as an androgen biosynthesis inhibitor that is currently approved in both pre- and postdocetaxel setting of mCRPC. The pivotal COU-301 study showed improvement in overall survival in the abiraterone with prednisone arm at 14.8 months versus 10.9 months in the prednisone only arm, with a 35% reduction in the risk of death in the abiraterone arm [15]. In addition, effective pain palliation and prevention of SREs have also been reported [12]. At a follow-up of 20 months, the median time to occurrence of first SRE was longer with abiraterone acetate and prednisone at 25 months compared to 20.3 months in the prednisone only arm. Similarly, abiraterone acetate and prednisone resulted in significantly more palliation in 157 of 349 (45.0%) of patients versus 47 of 163 (28.8%) in those patients with clinically significant pain at baseline. Notably faster palliation was achieved with abiraterone and prednisone with a median time to palliation of 5.6 months versus 13.7 months in those who did not receive abiraterone. In the COU-302 trial, abiraterone acetate plus prednisone before docetaxel was shown to yield a significant improvement in radiographic progression-free survival despite no improvement, though improved trend, towards overall survival [75]. 

### 5.4. Enzalutamide

Enzalutamide (formerly MDV3100, Xtandi, Astellas, Northbrook, IL; Medivation, San Francisco, CA) is an antiandrogen currently approved for postdocetaxel chemotherapy progression of mCRPC. The AFFIRM trial showed an improvement in overall survival in men who received enzalutamide with a median of 18.4 months versus 13.6 months in the placebo group [11]. While only a secondary endpoint, enzalutamide has also been shown to retard SREs with delayed time to the first SRE at 16.7 months versus 13.3 months in those who received placebo; hazard ratio, 0.69; *P* < 0.001). In addition, all parameters of pain palliation, including time to pain progression, mean reduction in pain intensity as well as reduction in pain interference were all in favor of the enzalutamide compared to the placebo arm [14].

## 6. Conclusion

The recent understanding of the molecular mechanisms of bone metastases in mCRPC has resulted in the significant development of new bone-targeted agents. Bone involvement in mCRPC is a source of significant morbidity including pain and SREs and targeting the bone microenvironment leads to improvement of quality of life, reduction of bone complications, and more recently, improvement in survival with a radiopharmaceutical. While specific bone-targeted agents have been approved and used routinely in practice as prevention of skeletal-related events, contemporary therapeutic agents that yield survival benefits in the form of novel androgen biosynthesis inhibitors or antiandrogens appear to have similar efficacy in delaying skeletal-related events (see Table 2 regarding effects on palliation and SREs of various agents), perhaps as a result of improved antitumor effects. The overarching question would be as follows: Is there a continued need for specific bone-targeted agents when contemporary drug therapies that have inherent antitumor, hence bone, effects achieve the same purpose? The field of prostate cancer therapy is rapidly evolving. As clinical trials start incorporating biomarker analyses with bone turnover markers and measuring specific bone-targeted endpoints, better understanding of the interplay between specific drugs to harness the benefits and obviate the side effects from these agents is becoming a reality in prostate cancer therapy.

## Figures and Tables

**Table 1:** Characteristics of selected FDA-approved bone targeting agents in mCRPC.

	Zoledronic acid	Denosumab	Sr-89	Sm 153	Ra 223
Class	Bisphosphonate	Monoclonal antibody against RANK-L	Pure Beta-emitter radiopharmaceutical	Beta and Gamma-emitter radiopharmaceutical	Alpha-emitter

Major side effects	Flu-like symptoms, hypocalcemia, osteonecrosis of the jaw	Hypocalcemia, osteonecrosis of the jaw	myelosuppression	myelosuppression	Nausea, vomiting, diarrhea.

Half-life (days)	6	25.4	50	1.9	11.4

Landmark randomized trial	Saad et al., 2002, 2004 [5, 6]	Fizzazi et al., 2011 [16]	Lewington et al., 1991 [17]	Serafini et al., 1998 [18] Sartor et al., 2004, 2007 [19, 20]	Parker et al., 2012 [21]
Arms	Zoledronic acid versus placebo (*n* = 643)	Denosumab versus zoledronic acid (*n* = 1940)	Sr-89 versus placebo (*n* = 32)	Sm 153 versus placebo (*n* = 118)	Ra 223 versus placebo (*n* = 922)
Endpoint	Significant decrease and delay in SREs and bone pain	Significant delay in SREs	Significant decrease in bone pain	Significant decrease in bone pain	Significant increase in OS, PSA drop

Status	FDA approved 2002	FDA approved 2010	FDA approved 1993	FDA approved 1997	FDA approved 2013

Administration	Intravenous	Subcutaneous	Intravenous	Intravenous	Intravenous

**Table 2:** The effect of selected agents on skeletal-related events (SREs) and pain palliation response based on randomized clinical trials.

Agent	SRE (% incidence or time to SRE)	Pain palliation response
Docetaxel versus mitoxantrone [8]	NE	35% versus 22% (*P* = 0.01)
Abiraterone acetate versus placebo [12, 13]	25.0 versus 20.3 months (*P* = 0.0001)	45% versus 28.8% (*P* = 0.005)
Enzalutamide versus placebo [11, 14]	16.7 versus 13.3 months (*P* < 0.0001)	NR
Cabazitaxel versus mitoxantrone [74]	NE	9.2% versus 7.7% (*P* = 0.63)
Zoledronic acid versus placebo [5]	33.2% versus 44.2% (*P* = 0.021), 14.9 months versus 10.7 months (*P* = 0.002)	−0.47% bone pain index (*P* = 0.024)
Denosumab versus zoledronic acid [16]	20.7 versus 17.1 months (*P* = 0.0008)	NE
Denosumab versus placebo (non-mCRPC) [44]	29.5 versus 25.3 months (*P* = 0.0028)	NE
Sm-153 versus placebo [18]	NE	72% pain relief (*P* < 0.034)
Sr-89 [53]	NE	Mean complete pain response 32%, mean partial pain response 44%
Ra 223 versus placebo [21]	15.6 versus 9.8 months (*P* = 0.00046)	NE
Cabozantinib [67]	NE	64% improvement

NE: not examined and NR: not reported.
